# Contrast Media Adverse Drug Reactions in Highly Polluted Environment

**DOI:** 10.3390/ijerph19127077

**Published:** 2022-06-09

**Authors:** Natalia Sauer, Wojciech Szlasa, Laura Jonderko, Krystyna Głowacka, Katarzyna Karłowicz-Bodalska, Anna Wiela-Hojeńska

**Affiliations:** 1 Faculty of Pharmacy, Wroclaw Medical University, 50-556 Wrocław, Poland; laura.jonderko@student.umw.edu.pl; 2 Faculty of Medicine, Wroclaw Medical University, 50-367 Wrocław, Poland; wojciech.szlasa@outlook.com; 3 Department of Clinical Pharmacology, Faculty of Pharmacy, Wroclaw Medical University, 50-367 Wrocław, Poland; krystyna.glowacka@umw.edu.pl (K.G.); anna.wiela-hojenska@umed.wroc.pl (A.W.-H.); 4 Department of Drugs Form Technology, Faculty of Pharmacy, Wroclaw Medical University, 50-556 Wroclaw, Poland

**Keywords:** iodinated contrast media, gadolinium contrast media, adverse drug reactions, contrast media, pollution

## Abstract

Iodinated- (ICM) and gadolinium-based (GCM) contrast media are used in radiology imaging techniques, such as computer tomography (CT) and magnetic resonance (MR), respectively. The paper aims to analyze the adverse drug reactions of ICM and GCM on different sites of the body in a highly polluted environment. We analyzed the pharmacovigilance in contrast media on the basis of reports submitted to the Regional Center for Monitoring of Adverse Drug Reactions (ADR) at the Department of Clinical Pharmacology in Wrocław. Safety profiles were compared between different ICM and GCM and at the system organ level using the proportional reporting ratio (PRR). We analyzed 124 reports of adverse reactions related to contrast agents between 2006 and 2021. Our findings revealed that ADR combinations occurred more frequently after the use of iodinated contrast agents (72.08%) than gadolinium contrast agents (27.92%). Iomeprol and Iopromide were identified as the most frequently reported media. Each medium presented a different safety profile. Skin disorders are the most common adverse drug reactions among patients using both iodine- and gadolinium-based contrast media. Gadolinium-based contrast agents are characterized by similar organ toxicity. Conversely, iodine-based contrast agents are more diverse—some of which show tissue specificity, such as Iodixanol for the gastrointestinal system or Iohexol for the respiratory tract. This study shows relatively high occurrence of respiratory tract related ADRs in Wrocław. We also prove that it is possible to choose the most optimal contrast agent for patients with specific organ site problems to omit the possible complications.

## 1. Introduction

Computer tomography (CT) and magnetic resonance (MR) in some cases require the use of contrast agents to enhance the visibility of specific structures [1]. The two main groups of contrast media remain iodine-based and gadolinium-based compounds. The first group finds its application in CT imaging and the latter in MR techniques. Iomeprol, Iopromide, Ioversol, Iodixanol, and Iohexol are the examples of iodine contrast media [2]. Conversely, Gadobutrol, Gadobenic acid, Gadoteridol, and Gadoteric acid are the examples of Gadolinium contrast media [3]. Osmolarity remains an important trait in case of contrast media adverse drug reactions. Iomeprol, Iopromide, Ioversol, and Iohexol are characterized by low osmolarity, whereas Iodixanol is iso-osmolar [4]. On the other hand, the osmolality of Gadoteridol (ProHance) is the lowest (0.63 Os/kg) of all the analyzed GCM. The osmolality of other specimens remains relatively higher, being 1.6 Os/kg for Gadobutrol (Gadovist), 1.97 Os/kg for Gadobenic acid (MultiHance), and 1.4 Os/kg for 0.5 M solution of Gadoteric acid (Dotarem) [5].

**Figure 1:**
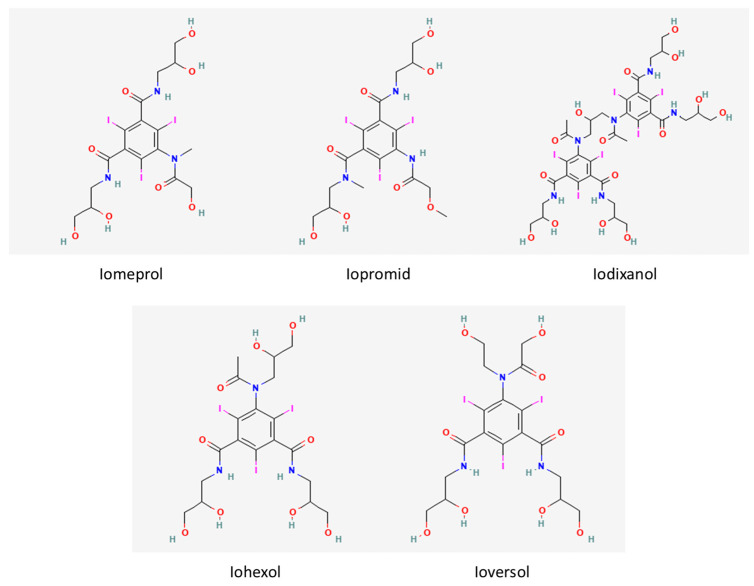
Iodine-based contrast media.

The chemical structure of Iomeprol and Iopromide differs in the position of a single methyl group. Namely, Iopromide is an ester and Iomeprol is a triamine. Both Ioversol and Iohexol are amides. Aside from Iodixanol, which is a dimer, all the mentioned compounds have three iodine atoms attached to the aromatic benzene ring (Figure 1).

**Figure 2:**
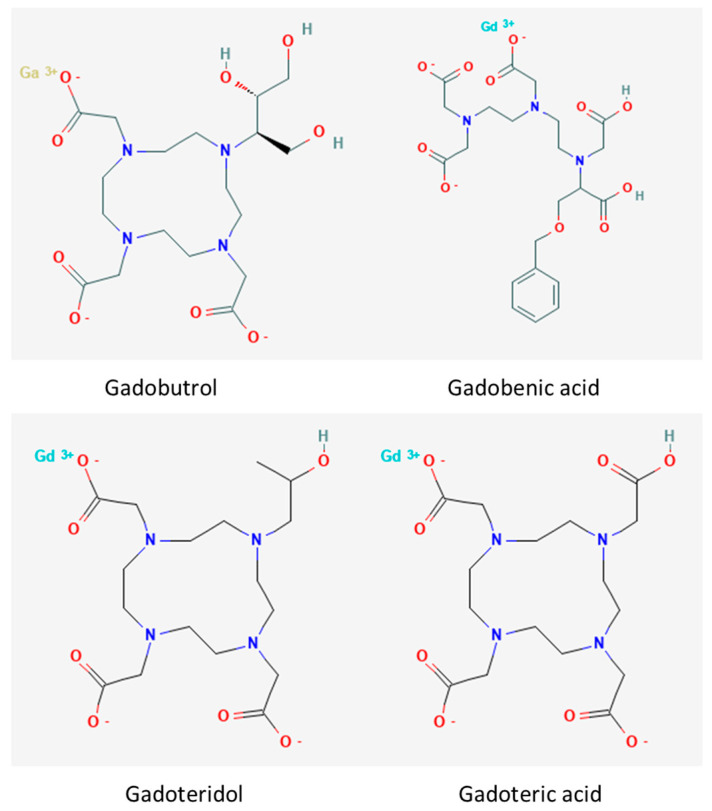
Gadolinium−based contrast media.

Gadolinium contrast media are mostly macrocyclic compounds, with the gadolinium (3+) ion in the core of the complex. Conversely, Gadobenic acid is an example of a chelate that does not contain a macrocyclic ring. Contrast media such as Gadobutrol, Gadoteridol, and Gadoteric acid are composed of gadolinium ion in the center, chelated by nitrogen from triamine groupings and side chains of the residues (Figure 2).

Most of the adverse drug reactions related to the use of gadolinium contrast media can be qualified as mild. These include pain in the joints, head, skin, and chest. The others are vomiting and difficulty breathing [6,7]. Gadolinium retention is responsible for the delay in the occurrence of adverse drug reactions after an MRI scan [3]. Namely, the element builds itself into the bones, brain, and kidneys and stays there for years. Therefore, its release may be delayed in time. Depending on the structure of the gadolinium compound, the effects may be more or less prominent. Linear agents are more likely to release the free gadolinium (3+) ion than the macrocyclic agents, thus the toxicity is mostly related with the application of macrocyclic gadolinium agents [3]. Several attempts were made to decrease the number of adverse drug reactions related to gadolinium contrast media [8], one of which included the application of chelating agents that bind the gadolinium complexes and enhance its release through the kidneys. All the compounds require high clinical susceptibility due to the variety of adverse drug reactions after their application. Iodine-based contrast agents may induce mild reactions, such as skin rash, urticaria, flushing, headache, itching, or nausea. Further, moderate reactions include wheezing, arrhythmias, hypertension, and difficulty in breathing. The most severe ones may be cardiac arrest, convulsions, hypotension, or swelling of the throat. In addition, when treating patients with kidney impairment, doctors should assess the benefits of contrast administration against any risks [9]. Patients suffering from heart, respiratory, or haematological disorders should be appropriately qualified for the use of iodine contrast media [10,11]. Additionally, patients treated with non-steroidal anti-inflammatory drugs (NSAID), beta-blockers, or interleukin 2 (IL-2) are disqualified from iodine contrast CT [12]. Even though the interactions between contrast media agents were already described, the effect of air pollution on the occurrence of ADRs has not been analyzed to date. For the study, we have chosen Wrocław—a highly polluted city in the west of Poland [13,14].

The paper aims to analyze the adverse drug reactions of iodine- and gadolinium-based contrast media on different sites of the body. We analyzed the data from the Regional Centre of Adverse Drug Reactions (ADRs) monitoring in Wrocław (Poland). The single center and the same studied population allowed us to compare agents used in both imaging techniques in case of inducing site-specific disorders. This approach allows clinicians to choose the most optimal imaging technique for patients with specific disorders. Furthermore, we compared the contrast media drugs based on their adverse drug reactions. The study presents differences in the occurrence of organ-site related adverse drug reactions depending on the contrast media, used both in CT and MR imaging techniques. Due to the problems with the pollution in Wrocław, the study is the first to compare both iodine- and gadolinium-based contrast media related ADRs in a highly polluted environment.

## 2. Materials and Methods

### 2.1. Data Source

This study used the database of spontaneous reports of ADRs from the Regional Centre of ADRs monitoring in Wroclaw (RCMADR). We obtained all reports of ADRs submitted to the RCMADR between 12 March 2006 and 21 September 2021. Each report contains information about patient demographics, the profession of the person reporting (specialty physician, pharmacist, nurse, or paramedic), route of administration (oral, intravenous, intramuscular, or external), ADRs, suspected drugs and concomitant drugs, patient outcomes, results of causality assessment, and report centers. Reported diagnoses were coded using the International Classification of Disease (ICD-10). ADRs were coded according to the Medical Dictionary for Regulatory Activities (MedDRA) terminology and verbatim drug names were coded to extract standardized generic names according to the Anatomical Therapeutic Chemical (ATC) classification. We analyzed the short-term adverse reactions after the administration of the contrast medium, which were observed during the CT or MR imaging and in the observation period after the medical procedure. Long-term effects were not included in the study nor reported by the physicians.

### 2.2. Patients Group

The study was performed on the total group of 124 patient reports—80 females (64.52%) and 44 males (35.48%). The patients had a mean age of 46.92 (standard deviation, 18.62). The characteristics of the study group are shown in Table 1.

### 2.3. Statistical Analysis

A single report can contain more than one suspected drug or more than one adverse effect, though a report containing two ADRs with one contrast medium was counted as two ADRs. For the analysis, all the reported ADRs of iodine- and gadolinium-based contrast media were listed. We analyzed the data at the level of reports, patient demographics (age, gender), and the presence of ADRs for each contrast medium. The safety profile was characterized and compared between iodine- and gadolinium-based compounds. For this analysis, ADRs were grouped into the primary System Organ Class (SOC) in the WHO Adverse Reactions Terminology (WHOART) and the frequency of ADRs was calculated. The proportional reporting ratio (PRR) was calculated to compare the safety profiles of each individual iodine- and gadolinium-based contrast medium. The PRR is calculated by the ratio between the frequency with which a specific adverse event is reported for the contrast medium and the frequency with which the same adverse event is reported for all contrast media in the comparison group (Table 2) [15,16]. A PRR value greater than 1 suggests that the adverse event is more commonly reported for individuals taking the drug of interest, relative to the comparison drugs, and this drug could indicate an adverse event.

## 3. Results

**Figure 3:**
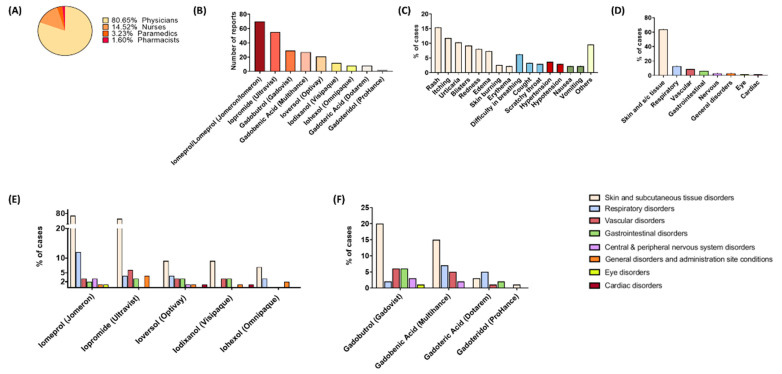
(**A**) Percentage participation in reporting of the contrast media adverse drug reactions; (**B**) Number of reports for each of iodine- and gadolinium-based contrast media; (**C**) Percentage participation of specific adverse drug reactions in the group of reports; (**D**) Adverse drug reactions divided into organ-specific sections; (**E**) Detailed participation of each side effect in the toxicity of each iodine-based contrast medium; and (**F**) Detailed participation of each side effect in the toxicity of each gadolinium-based contrast medium.

We obtained 836 reports of ADRs submitted to the RCMADR between 12 March 2006 and 21 September 2021. The number of reports of adverse reactions related to contrast agents was 124, which is 14.83% of all reports. The other 712 reports related to the use of other drugs. In total, 91 of 124 (73.39%) reports were related to iodinated contrast media and 33 (26.61%) to gadolinium-based compounds (Table 3). Among the analyzed reports, 100 reports (80.65%) came from physicians, 18 (14.52%) from nurses, 4 from paramedics (3.23%), and 2 were sent by pharmacists (1.60%) (Figure 3A). Of a total of 283 ADR combinations, the most often reported ADRs at the SOC level were associated with skin and subcutaneous tissue disorders (63.96%, *n* = 181) (Figure 3C). The minority of ADRs were disorders of the central and peripheral nervous system (chills, swoon, seizures, and numbness), general disorders and administration site conditions (fever, anxiety, weakness, malaise, and hyperhidrosis), eye disorders (conjunctival hyperemia and epiphora), and cardiac disorders (sudden cardiac arrest and palpitations) (Figure 3D).

### 3.1. Gadolinium-Based Contrast Media

In total, 79 (27.92%) ADRs were related to gadolinium-based contrast media. Each medium presented a different safety profile. Among the 19 ADRs groups classified according to the SOC, there were six signals categorized as potentially causing adverse effects (Table 4). There were five detected signals of disproportionate reporting for Gadobutrol. Among ‘eye disorders’, there was only one reported ADR characterized as ‘epiphora’. The ‘respiratory disorders’ ADRs included three cases of ‘difficulty in breathing’, two cases of ‘cough’, and two cases of a ‘scratchy throat’. For the gadoteric acid and gadoteridol, there were no cases of disproportionate reporting (compare to Figure 3E).

### 3.2. Iodine-Based Contrast Media

Iodine-based contrast media accounted for 72.08% (*n* = 204) of the 283 ADR combinations (Table 5). There were four detected signals of disproportionate reporting for Iomeprol. For the signal of ‘eye disorders’, the number of ADR combinations totaled one case of ‘conjunctival hyperemia’. The signal of ‘eye disorders’ came from sudden cardiac arrest, and it was the only serious adverse event. The patient was female and 64 years old; she used Iomeron without other concomitant medication. Iopromide presented three signals of disproportionate reporting, the highest PRR value was for ‘general disorders and administration site conditions’ and included four ADR combinations. There were two cases of ‘fever’, one case of ‘hyperhidrosis’, and one case of ‘weaknesses’. For Ioveresol, there were seven detected signals of disproportionate reporting, with the highest PRR value for the signal of ‘cardiac disorders’. For the signal, the number of ADR combinations was one and included ‘palpitations’. Iodixanol presented four signals of disproportionate reporting. The signal of ‘gastrointestinal disorders’ presented the highest PRR value; the number of ADR combinations was two and included one case of ‘nausea’ and one case of ‘vomiting’. There were two detected signals of disproportionate reporting for Iohexol. For the signal of ‘general disorders and administration site conditions’, the number of ADR combinations was two for ‘fever’ and ‘anxiety’ (compare to Figure 3F).

### 3.3. Differences in the ADR Profiles between Individual Contrast Media

Each contrast medium presented a different safety profile. For the category ‘skin and subcutaneous tissue disorders’, the calculated PRR was the highest for the Gadobutrol (PRR = 1.12), followed by Iomeprol (PRR = 1.03) and Iopromide (PRR = 1.02). Among the ‘respiratory disorders’, the highest PRR value was observed for the Iohexol (PRR = 1.94), then Ioversol (PRR = 1.54), Gadobutrol (PRR = 1.13), and Iomeprol (PRR = 1.12). For the category ‘vascular disorders’, the calculated PRR was the highest for the Ioversol (PRR = 2.03), followed by Iopromide (PRR = 1.32); there was only one detected signal of gadolinium-based contrast medium, which came from Gadobutrol (PRR = 1.13). Similarly, for the category ‘gastrointestinal disorders’, only one detected signal derived from gadolinium contrast medium, which was Gadobutrol (PRR = 1.63); the highest PRR was for the Iodixanol (PRR = 2.99), followed by the Ioversol (PRR = 2.61). For the category ‘central and peripheral nervous system disorders’, there was also only one signal connected with gadolinium-based compounds, which came from Gadobutrol (PRR = 1.68); the highest PRR value was for Ioversol (PRR = 2.30), soon followed Iomeprol (PRR = 1.62). In the case of ‘general disorders and administration site conditions’, signals of disproportionate reporting were observed only in the group of iodinated contrast media. Iohexol was characterized by the highest PRR value (PRR = 3.30). The PRR value associated with other media were as follows: for Iodixanol, PRR = 1.78; Iopromide, PRR = 1.28; and Ioversol, PRR = 1.05. Among the ‘eye disorders’, there were two signals of disproportionate reporting from Gadobutrol (PRR = 2.23) and Iomeprol (PRR = 2.17). In the group of gadolinium-based contrast media, there were no detected signals for the category ‘cardiac disorders’, whereas in the Iodinated contrast media group signals were observed from Ioversol (PRR = 4.56) and Iomeprol (PRR = 1.09).

## 4. Discussion

In this study, the safety profile of iodine and gadolinium contrast agents in routine clinical use was evaluated. Although the use of contrast media is generally considered to be safe and beneficial in medical imaging, it occasionally results in adverse events in patients. Identified differences in ADR profiles by organ class were compared between contrast agents. The results were used to evaluate the toxicity of each CM at a specific organ site. Cutaneous symptoms were the most common clinical manifestation, followed far behind by respiratory and vascular symptoms. The adverse reactions usually begin within 20 min after the administration of the contrast media. The anaphylactoid reaction might be divided into three groups: severe (hypotension, arrythmias, death, edema, etc.), moderate (tachycardia, bradycardia, bronchospasm, etc.), or mild (rash, itching, nausea, etc.) [17,18]

Our findings revealed that ADR combinations occurred more frequently after the use of iodinated contrast agents (72.08%) than gadolinium contrast agents (27.92%). Comparable statistics were conducted by Hunt et al., where a total of 522 adverse effects were identified [19]. Four hundred and fifty-eight of these adverse effects were associated with low-osmolar iodinated contrast material (87.74% of all ADRs) and only sixty-four adverse effects were reported with gadolinium contrast material (12.26% of all ADRs). Gadolinium contrast agents were reported significantly less frequently and had a significantly lower rate of adverse reactions; the percentage for gadolinium contrast material was 0.04%, and 0.15% for iodinated contrast material.

Furthermore, of all CM reports in our database, reports on iodinated contrast agents represented the majority (73.39%) and reports on gadolinium contrast agents accounted for only 26.61%. Similar proportions of reports were registered in the World Health Organization Uppsala Monitoring Centre. Between 2006 and 2021, the number of reports on the same iodinated contrast agents reached 197,897 (84.03%), whereas the number of reports on gadolinium contrast agents was merely 37,599 (15.97%). There has been an increase in the number of reports in recent years, which may be explained as a result of the training of centers in pharmacovigilance and knowledge acquisition on adverse reactions to contrast agents by healthcare professionals [20,21].

‘Skin and subcutaneous tissue disorders’ were most common according to the CM-ADR reports received in our study, similar to previous reports [22,23,24]. The most frequent skin manifestations included rash, itching, and urticaria which are characteristics of emerging allergic reactions. This was an expected finding because hypersensitivity reactions have commonly occurred in patients receiving iodinated and gadolinium contrast media [25,26]. Previous findings showed that non-iodinated contrast agents have a safer profile compared to iodinated contrast media (ICM) with the incidence of immediate adverse reactions being very low for gadolinium-based contrast agents [7,27,28,29,30]. In several studies, low-osmolality (nonionic monomers, ionic dimers) and iso-osmolality contrast media (nonionic dimers) were estimated to be safer than ionic dimers and cause less dermatological disorders [31]. The incidence of adverse events has decreased considerably with the change of usage from high-osmolar contrast media to low-osmolar contrast media. Gomi et al. examined the proportion of patients experiencing adverse events after use of ICM (Iomeprol, 3.9%; Iopamidol, 2.2%; Iohexol, 2.0%; Iopromide, 3.5%; Ioversol, 1.8%; and all five combined, 2.7%) among the 8931 patients. A study published by Seong et al. summarized the safety of seven Iodinated contrast media, where for Iomeprol, the percentage of ADRs of ‘skin and appendages disorders’ was substantially higher [32]. Observations in our study are similar; the incidence of ADRs of dermatological disorders was significantly higher for Iomeprol in the ICM group. However, we cannot exclude the possibility that this may be a result of frequent use in patients. In the group of gadolinium-based contrast media, skin adverse reactions occurred more frequently in patients who received macrocyclic GBCA—Gadobutrol. These observations find confirmation in the study of McDonald et al. who compared reaction rates between GBCAs and demonstrated that Gadobutrol had higher rates of allergic-like reactions compared with patients administered other gadolinium-based contrast media [33]. We found that higher rates of immediate allergic adverse events are associated with the properties of ionicity, protein binding, and cyclic structure. Lastly, our results corroborate the recently published meta-analysis of nine independent GBCA allergic-like reaction studies by Behzadi et al. [34].

For Iomeprol, the percentage of ADRs of ‘respiratory system disorders’ was higher than that induced by other ICM (52.17%). A similar tendency was observed by An et al., who described the Iomeprol as most commonly resulting in ADRs; the prevalence of ‘respiratory system disorders’ such as bronchospasm was significantly higher than that induced by other ICM [35]. However, the ability to indicate the adverse event was the highest for Iohexol (PRR = 1.94). A study by Iyer et al. proved that using Iohexol decreases ventilatory functions to a small but significant extent in patients without any overt pulmonary disease [36]. The results following the use of gadolinium-based contrast agents revealed their safety profile; our findings suggest that only Gadobutrol could potentially be responsible for indicating pulmonary adverse events. Notable reported respiratory symptoms included respiratory distress, bronchospasm, and pulmonary edema [37,38,39,40,41,42,43]. Nevertheless, short-term adverse drug reactions are very rare (<2.5%), and the vast majority of them are minor, transient, and do not require treatment [44,45]. Gadolinium-based contrast agents are considered safe alternatives to iodine-based contrast agents, with a comparatively lower incidence of adverse effects.

In our study, the PRR points to the relative frequency of ‘vascular disorders’ for Ioversol, including hypertension and hypotension. Contrast agents are known to cause some changes in blood flow and blood pressure, although these are not thought to be clinically significant [46,47,48,49,50,51]. Singh et al. showed that the incidence of mild and moderate contrast reactions is higher for HOCM (high osmolar contrast media) (6–8%) than for LOCM (low osmolar contrast media) (0.2%) and anaphylactoid reactions are more common while using HOCM. Conversely, cardiovascular decompensation is more common while using LOCM [52].

Late adverse reactions after intravascular contrast medium include symptoms such as nausea, abdominal pain, diarrhea, and vomiting, with are the most common ‘gastrointestinal disorders’ [2]. Contrast media are known to be toxic to the gastrointestinal system, however our findings show different proportions in the frequency of their occurrence. A study by Kalaiselvan et al. suggests that gastrointestinal symptoms are the most commonly reported after the cutaneous symptoms [53]. Curiously, Morales-Cabeza et al. also presented similar tendencies; skin disorders were the most common clinical manifestation, followed by gastrointestinal issues. Conversely, our research revealed that these disorders are much less common; the reason may be the low quality of air in Wrocław, which leads to a prevalence of respiratory dysfunctions. Several human reports have shown that patients with lung diseases have more acute symptoms [30,54,55,56,57,58]. It is well known that environmental air pollution can increase the degree of airway hyperresponsiveness and consequently the risk of asthma exacerbations [59,60,61,62,63].

Contrast media rarely induce neurological complications. However, neurotoxicity is an important and dose-dependent effect, appearing to be caused by disruption of the blood–brain barrier by the high osmolarity of the contrast agent [64]. From devastating encephalopathy to numbness and seizures, an array of neurological manifestations has been described in the literature [65,66,67]. Hyperosmolarity of some contrast agents can disrupt the blood–brain barrier by drawing water from endothelial cells of brain capillaries, causing cell shrinkage and separation of tight junctions [68]. Diverse neurological sequel ADR of intravenous contrast administration have been reported in several case studies [69,70,71,72,73]. Although many studies have suggested that the risk of developing nervous system disorders is higher in response to high osmolality agents, our findings demonstrate that symptoms can also occur in response to small amounts of low-osmolar, non-ionic contrast agents. Nevertheless, the clinical significance of contrast media deposition in the brain remains unclear [74].

Patients with general disorders are often unaware of their presence and may not report symptoms, remaining undiagnosed. Thus, symptoms such as fever, anxiety, weakness, and malaise are likely to be under-reported and may be even a more prevalent than our data reflect. The incidence of serious adverse effects is low, and they are mainly late adverse reactions, which are defined as reactions occurring 1 hour to 1 week after contrast medium injection [2]. There remain major areas of uncertainty, and there is insufficient data in the existing literature to guide practice. Symptoms of general disorders were infrequently reported in our study and were not related to gadolinium contrast agents.

Cardiac and Ophthalmic adverse drug reactions are relatively unabundant. Cardiac disorders may be important in patients with severely impaired circulatory performance. Among the patients exhibiting delayed hypersensitivity reactions, only 2 (0.71%) patients showed cardiovascular symptoms, such as palpitations and sudden cardiac arrest. Three physicochemical properties determine the toxicity of ICM—osmolality, sodium concentration and calcium-binding capability [75]. Some studies suggest that the presence of comorbid cardiovascular disease may be a significant risk factor for adverse reactions [76,77]. A study by Akre et al. showed an advantage of Gadoteridol over gadopentate dimeglumine in cases of negative inotropic effect and ECG disturbances induction [78]. The incidence of ophthalmic adverse drug reactions may be correlated with the use of beta-blockers in ophthalmic preparations. The patients are more likely to exert an anaphylaxis reaction [54].

More and more studies report the newly found adverse drug reactions of the already known contrast media. For instance, due to the long accumulation time, levels of GCM stay high in tissues such as the brain, skin, and bones. In recent years, nephrogenic systemic fibrosis was observed as a novel side effect [44]. Therefore, much focus must be set on the monitoring of contrast media adverse effects.

## 5. Conclusions

Skin disorders, such as rash, itching, and urticaria are the most common adverse drug reactions among patients using both iodine- and gadolinium-based contrast media. Gadolinium-based contrast agents are characterized by similar organ toxicity. Conversely, iodine-based contrast agents are more diverse, some of which show tissue specificity, such as Iodixanol for the gastrointestinal system or Iohexol for the respiratory tract. Even though some drugs were reported to cause more adverse drug reactions than others, clinicians should consider the differences in the prevalence of their application. Our study revealed that the incidence of adverse drug reactions to contrast agents is different in highly polluted environments. Patients are significantly more prone to airway hyperresponsiveness after administration of iodinated and gadolinium contrast agents, and consequently the risk of respiratory distress increases. This study shows that it is possible to choose the most optimal contrast agent for patients with specific organ-site problems to omit the possible complications. Moreover, the study encourages physicians and pharmacists to report the newly diagnosed adverse drug reactions after the administration of contrast media.

## Figures and Tables

**Table 1:** Characteristics of patients group.

Age Range	Number of Patients	The Percentage of Reports in the Database
<18	7	5.65%
19–45	48	38.71%
46–60	26	20.97%
61–75	37	29.84%
76–90	4	3.23%
Unknown age	2	1.61%
Total	124	100%

**Table 2:** A table for a contrast medium and group of ADR in spontaneously reported data. Proportional reporting ratio (PRR) = [a/(a + b)]/[c/(c + d)].

	Specified Group (SOC) of ADRs	All Other Groups (SOCs) of ADRs	Total
Contrast medium of interest	a	b	a + b
All other contrast media	c	d	c + d
Total	a + c	b + d	a + b + c + d

**Table 3:** Frequency of reports and ADRs for each individual contrast medium (% values rounded to two decimal places).

Contrast Media	Total Reports		ADR	
	N	%	N	%
Iomeprol (Jomeron)	44	35.48	91	32.16
Iopromide (Ultravist)	31	25.00	67	23.67
Ioversol (Optivay)	7	5.65	22	7.77
Iodixanol (Visipaque)	5	4.03	12	4.24
Iohexol (Omnipaque)	4	3.23	12	4.24
Gadobutrol (Gadovist)	15	12.10	38	13.43
Gadobenic Acid (Multihance)	10	8.06	29	10.25
Gadoteric Acid (Dotarem)	7	5.64	11	3.89
Gadoteridol (Prohance)	1	0.81	1	0.35
Total	124	100	283	100

**Table 4:** Proportional reporting ratio of gadolinium-based contrast media.

Contrast Media	System Organ Class	PRR	% of ADRs
Gadobutrol (Gadovist)	Skin and subcutaneous tissue disorders	1.12	51.28%
	Respiratory disorders	0.37	14.29%
	Vascular disorders	1.13	50.00%
	Gastrointestinal disorders	1.63	75.00%
	Central & peripheral nervous system disorders	1.68	60.00%
	Eye disorders	2.23	100%
Gadobenic Acid (Multihance)	Skin and subcutaneous tissue disorders	0.93	38.46%
	Respiratory disorders	1.13	50.00%
	Vascular disorders	0.97	41.67%
	Central & peripheral nervous system disorders	0.93	40.00%
Gadoteric Acid (Dotarem)	Skin and subcutaneous tissue disorders	0.25	7.69%
	Respiratory disorders	0.85	35.71%
	Vascular disorders	0.21	8.33%
	Gastrointestinal disorders	0.60	25.00%
Gadoteridol (Prohance)	Skin and subcutaneous tissue disorders	0.09	2.56%

**Table 5:** Proportional reporting ratio of iodinated contrast media; * presents a single report.

Contrast Media	System Organ Class	PRR	% of ADRs
Iomeprol (Iomeron)	Skin and subcutaneous tissue disorders	1.03	47.89%
	Respiratory disorders	1.12	52.17%
	Vascular disorders	0.52	23.08%
	Gastrointestinal disorders	0.45	20.00%
	Central & peripheral nervous system disorders	1.62	75.00%
	General disorders and administration site conditions	0.25	11.11%
	Eye disorders	2.17	100.00% *
	Cardiac disorders	1.09	50.00%
Iopromide (Ultravist)	Skin and subcutaneous tissue disorders	1.02	35.21%
	Respiratory disorders	0.54	17.39%
	Vascular disorders	1.32	46.15%
	Gastrointestinal disorders	0.88	30.00%
	General disorders and administration site conditions	1.28	44.44%
Ioversol (Optivay)	Skin and subcutaneous tissue disorders	0.72	6.34%
	Respiratory disorders	1.54	17.39%
	Vascular disorders	2.03	23.08%
	Gastrointestinal disorders	2.61	30.00%
	Central & peripheral nervous system disorders	2.30	25.00%
	General disorders and administration site conditions	1.05	11.11%
	Cardiac disorders	4.56	50.00%
Iodixanol (Visipaque)	Skin and subcutaneous tissue disorders	0.96	5.63%
	Vascular disorders	1.25	7.69%
	Gastrointestinal disorders	2.99	20.00%
	General disorders and administration site conditions	1.78	11.11%
Iohexol (Omnipaque)	Skin and subcutaneous tissue disorders	0.88	4.93%
	Respiratory disorders	1.94	13.04%
	General disorders and administration site conditions	3.30	22.22%

## Data Availability

Not applicable.
